# Gut mycobiome alterations and implications for liver diseases

**DOI:** 10.1371/journal.ppat.1012377

**Published:** 2024-08-08

**Authors:** Suling Zeng, Bernd Schnabl

**Affiliations:** 1 Institute of Health and Medicine, Hefei Comprehensive National Science Center, Hefei, China; 2 Department of Medicine, University of California San Diego, La Jolla, California, United States of America; 3 Department of Medicine, VA San Diego Healthcare System, San Diego, California, United States of America; Universitat Zurich, SWITZERLAND

## Abstract

Chronic liver disease and its complications are a significant global health burden. Changes in fungal communities (mycobiome), an integral component of the gut microbiome, are associated with and contribute to the development of liver disease. Fungal dysbiosis can induce intestinal barrier dysfunction and allow fungal products to translocate to the liver causing progression of disease. This review explores recent progress in understanding the compositional and functional diversity of gut mycobiome signatures across different liver diseases. It delves into causative connections between gut fungi and liver diseases. We emphasize the significance of fungal translocation, with a particular focus on fungal-derived metabolites and immune cells induced by fungi, as key contributors to liver disease. Furthermore, we review the potential impact of the intrahepatic mycobiome on the progression of liver diseases.

## Introduction

**Fig 1:**
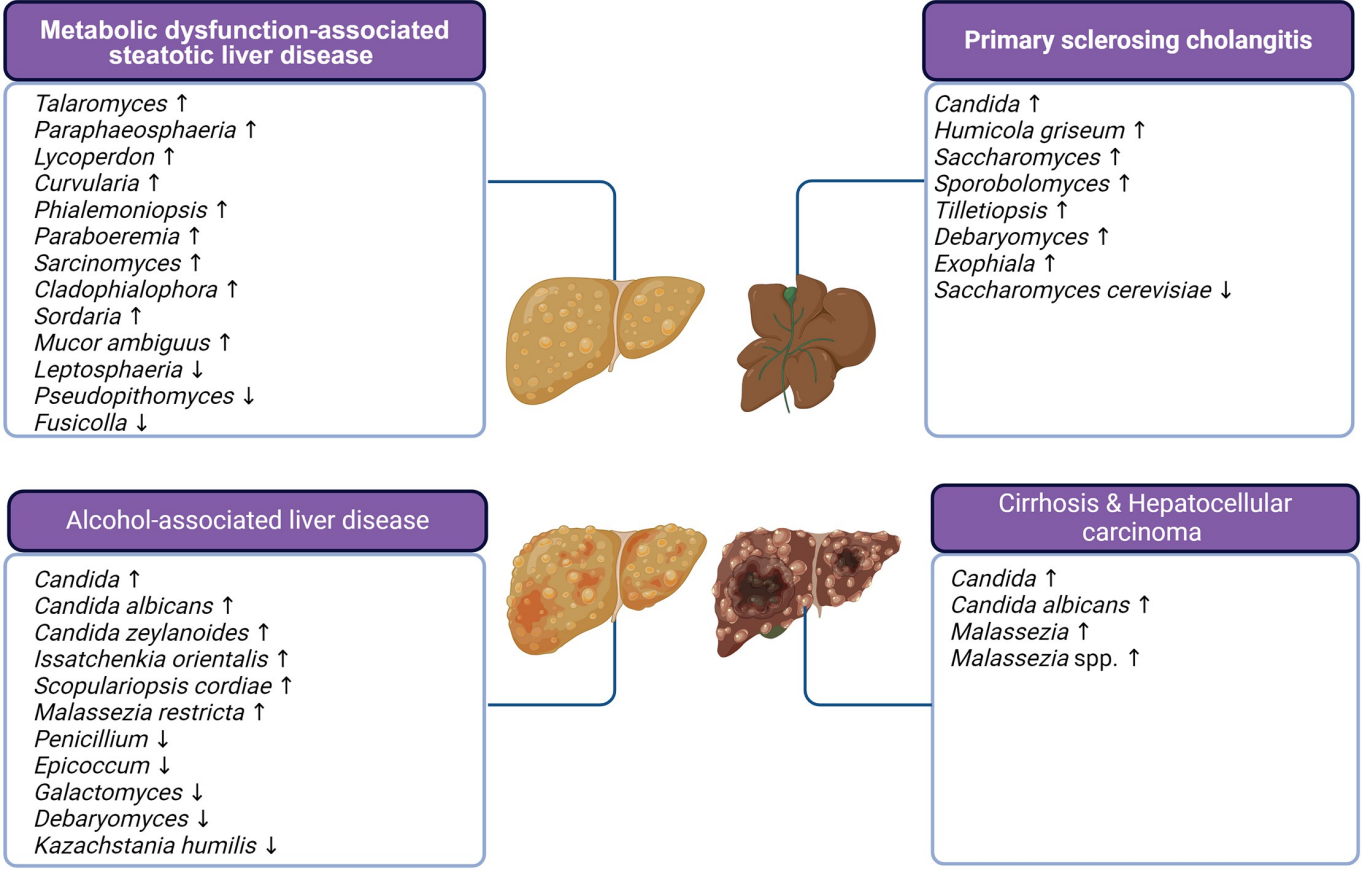
Alterations of gut mycobiome in liver disease. Created with a license from Biorender.com.

The gut and liver exhibit both functional and anatomical interconnectedness, facilitating the absorption of nutrients while restricting the systemic spread of microbes and toxins [1]. Within the gut microbiota, comprising bacteria, fungi, viruses, and archaea, the fungal component, or mycobiome, constitutes a small yet increasingly recognized fraction (less than 1%) with significant implications for intestinal homeostasis and liver health [2]. This mycobiome is now recognized as an important factor for the normal physiology of the liver and for the onset and progression of liver diseases [3,4]. In the past decade, changes in the composition of the gut mycobiome were characterized in healthy subjects and patient cohorts [5,6]. Utilizing both culture-dependent and independent methods, such as internal transcribed spacer (ITS) and 18S rRNA sequencing, alterations in mycobiome composition have been described across a spectrum of liver diseases, including metabolic dysfunction-associated steatotic liver disease (MASLD; formerly known as non-alcoholic fatty liver disease or NAFLD), alcohol-associated liver diseases, primary sclerosing cholangitis (PSC), cirrhosis, and hepatocellular carcinoma [7]. Correlation analyses have been employed to establish connections between disease severity and specific fungal species. To gain a better mechanistic understanding of how intestinal fungi affect the progression of liver disease, joint analyses of multi-omics data and mechanistic experiments in animals and cells have been employed to identify key contributors among fungal species. Given that the liver receives about 75% of its blood supply from the portal vein draining the intestine, intestinal fungi can impact liver biology remotely by secretion of metabolites and migration of immune cells. In this review, we summarize alterations of the gut mycobiome in patients with various liver diseases (Fig 1 and S1 Table) and describe how fungi contribute to liver disease. We also discuss the potential role of the intrahepatic mycobiome in the development of liver disease. Although this field is still in its early stages, the evolving understanding of the gut mycobiome in liver pathophysiology, spanning basic, translational, and clinical knowledge, offers promising avenues for fungi-targeted interventions to enhance outcomes in liver disease.

## Landscape of the gut mycobiome

Our knowledge of the structure and function of gut fungal communities is increasing rapidly due to high-throughput sequencing and computational advancements [8]. Fecal samples are analyzed by 18S, ITS1, ITS2 sequencing as well as whole-genome shotgun sequencing to profile signatures of the gut mycobiota. It is important to note that amplicon sequence-based analysis only shows relative abundances of species while not taking the absolute number of microbes into account. The absolute abundance of mycobiota can be determined by qPCR or culturomics [9]. In terms of the spatial dimension, the physiology of the intestine undergoes significant variation along its length from the duodenum to the colon. Similarly, the distinct biogeography of gut mycobiota profoundly influences fungal–host interactions [10]. Moreover, the distance from the intestinal mucosa introduces another dimension. Luminal and mucosal fungal communities cluster based on whether the samples were taken from luminal or mucosal sites rather than their longitudinal locations [10]. Mucosal fungi promote gut barrier function and social behavior via Th17 immunity [10], while luminal mycobiota are more affected by the environment and diet, thus might be transient in the feces. Therefore, the investigation of pathophysiological changes of site-specific mucosal gut mycobiota may provide new insights in host–microbial interactions.

The gut mycobiota can be detected in newborns after birth, then develop over time and mature into a relatively stable adulthood-like community with substantially increased diversity [11]. In 2017, the large-scale Human Microbiome Project (HMP) investigated the healthy human gut mycobiome by sequencing the ITS2 region and 18S rRNA gene in 317 samples from 147 volunteers [6]. *Ascomycota*, *Basidiomycota*, *Zygomycota*, and *Chytridiomycota* are the 4 main phyla of fungal communities [6]. The most abundant genera in the healthy adult gut mycobiome are *Candida*, *Saccharomyces*, and *Cladosporium*. *S*. *cerevisiae*, *M*. *restricta*, and *C*. *albicans* are the most detected species in the healthy human gut. The majority of detected species are highly diverse and not present in all healthy humans [12]. Therefore, researchers are making efforts to identify a common set of stable fungi—a core mycobiome. Fungal dysbiosis may be caused or induced by impaired functionality of the core mycobiome, which is associated with or in some cases, leads to disease. To simplify the global gut mycobiome variation into a few categories, dimensionality reduction efforts have been made to identify the core human mycobiome. Recent insights characterized 4 enterotypes of the human gut mycobiome from 3,363 fungal ITS sequencing samples from 16 cohorts across 3 continents: there is a *Saccharomyces*-dominated enterotype; a *Candida*-dominated enterotype; a *Aspergillus*-dominated enterotype; and an unclassified *Ascomycota* spp. and *Saccharomycetales* spp. dominated enterotype [5]. The 4 enterotypes show stable compositional patterns across populations, geographical locations, and correlate with bacterial enterotypes. Age is the major factor that affects fungal enterotypes. The *Candida*-dominated enterotype is enriched in elderly subjects, while the *Saccharomyces*-dominated and *Aspergillus*-dominated enterotypes are mostly enriched in the young participants. Moreover, the *Candida*-dominated enterotype is more prevalent in patients with disease [5]. This observation is consistent with the fact that *Candida*, one of the driver genus of the *Candida*-dominated enterotype, is arguably the most studied pathogenic fungus genera and is responsible for a variety of disease conditions [13].

In addition to host factors, the gut mycobiome can also be affected by dietary and environmental factors such as geography and urbanization. A recent shotgun metagenomic sequencing analysis of the fecal mycobiome from 942 healthy individuals across different geographic regions in China shows that urbanization-related factors had the strongest impact on gut mycobiome variation, followed by geography, dietary habits, and ethnicity [14]. Highly urbanized populations had a significantly lower fungal richness [14]. *Saccharomyces cerevisiae* was the only species enriched in urban participants compared with rural populations that inversely correlated with liver pathology-associated blood parameters. Based on the large-scale association analyses, more efforts are needed to move from descriptive mycobiota census analyses to cause-and-effect studies of the identified gut fungi in the pathogenesis of liver diseases.

## Changes of the gut mycobiome in patients with liver disease

### Metabolic dysfunction-associated steatotic liver disease (MASLD)

MASLD refers to a wide spectrum of liver disease ranging from simple hepatic steatosis and steatohepatitis to advanced fibrosis and cirrhosis [15]. The pathophysiology of MASLD is characterized with increased de novo synthesis of fatty acids, accumulation of lipids and elevated liver inflammation, resulting from genetic predisposition, lifestyle factors, and metabolic abnormalities. Most of gut mycobiome studies are descriptive studies on fecal fungal communities in patients with NAFLD from different regions. In China, fungal diversity was significantly increased in patients with NAFLD compared with matched healthy subjects as assessed by ITS2 sequencing [16] but decreased when metagenomic sequencing was used [17]. In a German cohort, however, no significant differences were detected [18]. At the genus level, *Cladophialophora* and *Sordaria* correlate with MASLD-related clinical parameters such as liver damage (e.g., ALT, GGT, AST) and lipid metabolism (e.g., total cholesterol and triglycerides) [16]. While at the species level, high enrichment of *C*. *albicans* and *Mucor* spp. were observed in patients with NAFLD [18]. Notably, this observation was shown in non-obese patients with NAFLD, for which risk factors are not well understood [18]. In line with this study, fecal *Mucor* species (*M*. *ambiguus*) enriched in MAFLD patients correlated with the lipid metabolism index (total cholesterol, low-density lipoproteins (LDL)) [17]. More interestingly, this study identified *M*. *ambiguus* as biomarker in both the oral and gut mycobiome, suggesting a potential oral-gut route of *M*. *ambiguous* in patients with MAFLD. Few studies have examined potential fungal therapeutic approaches for MASLD. Mice that were microbiota humanized with feces from patients with non-alcoholic steatohepatitis (NASH) and subjected to western diet feeding had reduced steatohepatitis with concomitant antifungal treatment using amphotericin B [18].

Future studies are needed to elucidate the role of specific fungi (e.g., *Mucor* sp.) for pathogenesis of MASLD. Similar to gut bacteria, fungi can possibly influence the metabolism of dietary nutrients, resulting in increased production of bioactive metabolites such as short-chain fatty acids (SCFAs), lipopolysaccharides (LPS), bile acids, and trimethylamine-N-oxide (TMAO), which can promote inflammation, adipokine production, and insulin resistance, contributing to MASLD development [19]. A recent study using mass spectrometry-based tools revealed vastly underappreciated modification of bile acids and related steroidal lipids that is regulated by host and microbial co-metabolism [20]. Fungi (e.g., Penicillium) can also bio-produce bile acids and the glycine conjugates, although the contribution of fungal bile acid derivatives to the development of MASLD is currently unknown [21].

### Alcohol-associated liver disease

Frequent and excessive alcohol misuse causes a spectrum of alcohol-associated liver diseases, including simple steatosis, steatohepatitis, fibrosis, and cirrhosis. Patients with underlying chronic alcohol-associated liver disease can develop acute-on-chronic alcohol-associated hepatitis, characterized by cholestasis and high mortality [1]. The main factors contributing to alcohol-associated liver disease include alcohol metabolism-induced acetaldehyde toxicity, oxidative stress, inflammation, and steatosis. Involvement of specific fungal members in the pathogenesis of alcohol-associated liver disease has been more studied compared with other liver diseases. Fungal species richness and diversity were consistently lower in patients with alcohol use disorder and alcohol-associated hepatitis as compared with healthy controls [22,23]. In patients with alcohol user disorder and alcohol-associated hepatitis, relative abundance of *Candida* was increased as compared with healthy subjects [23,24] and significantly decreased after 2 weeks of alcohol abstinence in fecal samples analyzed by ITS2 sequencing [24], which is also mirrored by lower serum anti-*C*. *albicans* immunoglobulin G (IgG) and M (IgM) levels. These studies consistently identified a potential role of *Candida* genus for the development of alcohol-associated liver disease [22,24]. Therefore, several studies have investigated the causal relationship between *C*. *albicans* and alcohol-associated liver disease. Colonized mice with *C*. *albicans* promoted ethanol-induced liver disease, while reducing intestinal fungi attenuated ethanol-induced liver disease in mice [25]. In addition to *C*. *albicans*, the presence of *Malassezia restricta* was also found to be associated with increased markers of liver injury in patients with alcohol use disorder [26]. *M*. *restricta* exacerbated ethanol-induced liver injury both in acute binge and chronic ethanol-feeding models in mice by inducing inflammatory cytokines and chemokines in Kupffer cells through C-type lectin domain family 4, member N (Clec4n, also known as dectin-2) signaling. More precise strategies to target specific fungi should be developed for alcohol-associated liver disease.

### Primary sclerosing cholangitis

PSC is a chronic liver disease characterized by inflammation and scarring of the bile ducts, which is in most cases accompanied by inflammatory bowel disease (IBD), particularly ulcerative colitis [27]. The exact cause of PSC is not fully understood, but autoimmune factors, genetic predisposition, bile duct injury, and inflammation are believed to contribute to its development. To distinguish mycobiota changes between those associated with PSC or resulting from comorbid conditions of IBD, the fecal mycobiome analysis of PSC patients is usually performed in cohorts comprising healthy controls, patients with PSC, patients with PSC and concomitant IBD, and patients with IBD. In both, a German [28] and an Italian pediatric cohort [29], fungal alpha diversity showed no significant difference in healthy controls, patients with PSC (with or without IBD), or patients with ulcerative colitis, while it was increased in PSC patients (with or without IBD) compared with healthy controls in a French cohort [30]. Compared with healthy controls and patients with ulcerative colitis, increased levels of the genera *Exophiala*, and a decreased proportion of *Saccharomyces cerevisiae* were observed in patients with PSC [30]. While in the pediatric PSC-IBD cohort [29], the relative abundances of *Saccharomyces*, *Sporobolomyces*, *Tilletiopsis*, and *Debaryomyces* were increased, and of *Meyerozyma* and *Malassezia* were decreased compared with healthy controls measured by ITS2 sequencing. Further studies are needed to investigate the causal relationship of specific gut fungi and PSC.

Interestingly, bacterial and fungal microbiota interactions were reported in the progression of PSC determined by 16S and ITS2 sequencing, respectively [30]. Co-occurrence analysis showed less nodes and edges for fungi networks compared with bacteria. Correlation network analysis demonstrated that PSC (with or without IBD) groups exhibited a lower fungi–bacteria network than healthy controls and IBD groups [30]. But it is very difficult to identify functionally important interactions between fungi and bacteria as the direct comparison of prokaryotic (bacterial, 16S) and eukaryotic (fungi, ITS) members of the microbiome using different amplicons for each kingdom is hindered. Multi-kingdom spike sequencing (MK-SpikeSeq) [31] has been developed to solve this problem by spiking a defined community of bacteria and fungi as an internal control. Future studies would benefit from MK-SpikeSeq to reveal previously uncharacterized fungi–bacteria interactions that influence microbiota composition and function in liver disease.

### Cirrhosis and hepatocellular carcinoma

Cirrhosis is the end stage of chronic liver disease and hepatocellular carcinoma (HCC) is arising predominantly in patients with cirrhosis. Different etiologies of chronic liver disease lead to cirrhosis including hepatic viral infections, alcohol misuse, metabolic syndrome, and hepatic toxin exposure. Fungal alpha diversity was lower in patients with cirrhosis without HCC [32] and HCC-cirrhosis [33] compared with healthy controls. An impaired immune system contributes to the occurrence of bacterial and fungal infections in patients with cirrhosis. Therefore, antibiotics and proton pump inhibitors are often used in these patients. Broad-spectrum antibiotics not only disrupted bacterial, but also fungal populations and decreased fungal diversity, while proton pump inhibitor treatment did not significantly affect fungal diversity in patients with cirrhosis or healthy controls [32]. Higher relative abundance of *Candida* was observed in the fecal samples from patients with cirrhosis using ITS1 sequencing [32], which was validated in another cohort using PCR-based and culture-dependent methods [34]. Translocation of fungal products, as measured by serum 1,3-β-D-glucan, correlated with severity and outcome in patients with cirrhosis [35]. With regards to the fungi–bacteria interactions, rich and complex correlations between fungi and bacteria were observed in the linkage patterns of healthy controls while they were reduced to a skewed linkage pattern in patients with cirrhosis [32]. A combined bacterial–fungal dysbiosis metric-Bacteroidetes/Ascomycota, proved to be a simple index to independently predict 90-day hospitalizations in patients with cirrhosis [32]. This observation highlights the diagnostic and prognostic role of fungi–bacteria interaction in patients with cirrhosis.

In patients with HCC, decreased fungal diversity was observed as compared with healthy controls or patients with cirrhosis [33]. Increased relative abundances of fecal *Malassezia* and *Candida* species were seen in patients with HCC compared with healthy controls and patients with cirrhosis by ITS2 sequencing [33]. Two independent studies validated that colonization of *C*. *albicans* promotes tumor progression in Hepa1-6 cells induced HCC model in mice [33,36]. The hepatocarcinogenesis effect of *C*. *albicans* may be linked to the up-regulation of NLRP6 [36]. In addition to the association of live intestinal fungi with the development of HCC, secondary metabolites of fungi, especially aflatoxins have been widely studied as strong inducers of HCC. A large-scale analysis of 348 patients with HCC and 597 healthy subjects revealed that exposure to aflatoxin significantly increased the risk of HCC development [37].

### Fungal translocation in liver diseases

**Fig 2:**
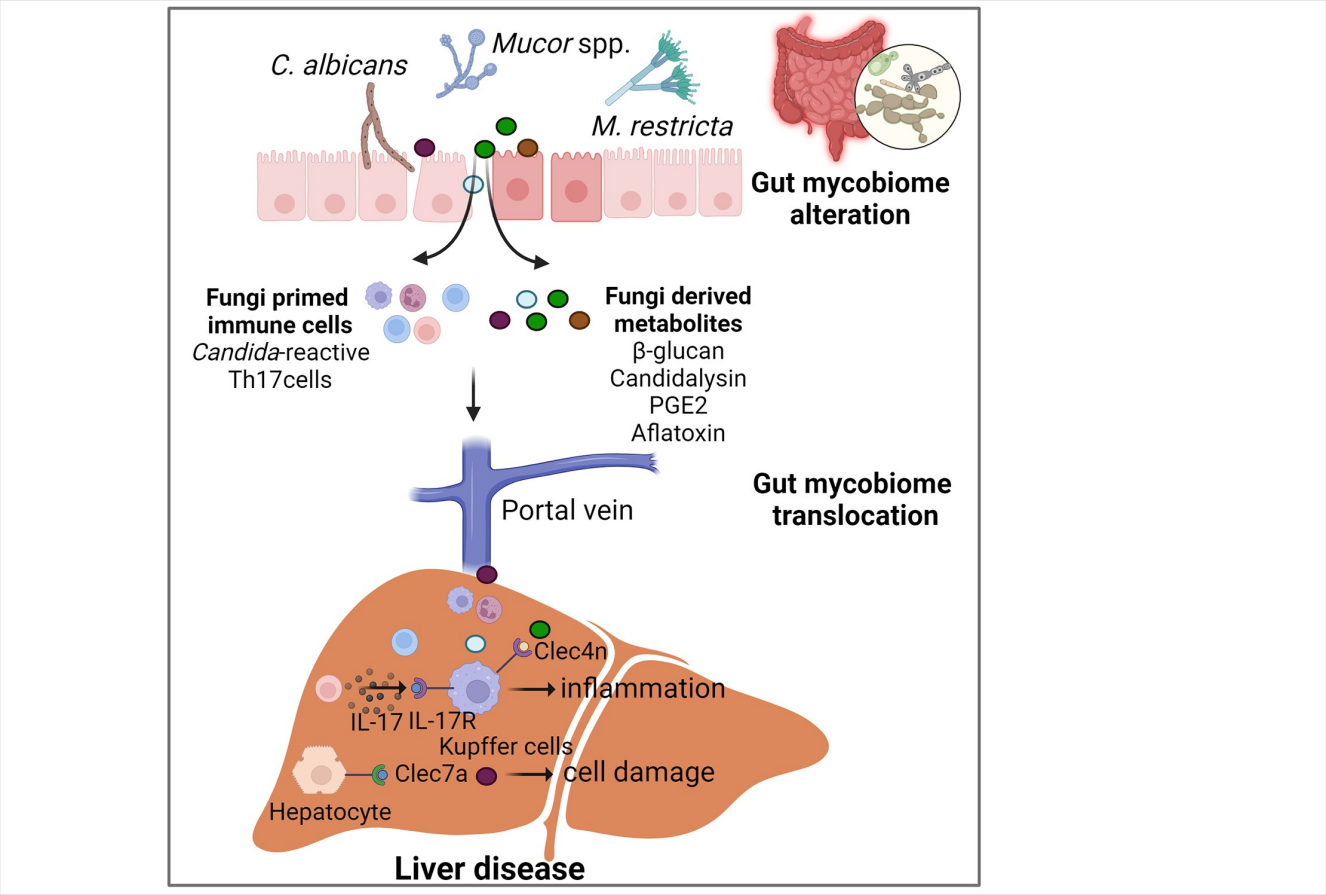
Translocation of gut mycobiome in liver disease. Chronic liver diseases are associated with gut fungal dysbiosis. Fungi-derived metabolites or fungi-primed immune cells can translocate to the liver and contribute to liver disease progression by enhancing inflammation or increasing hepatocyte damage. PGE2, prostaglandin E2; Clec7a, C-type lectin domain family 7 member A. **Created with a license from Biorender.com**.

Fungi resident in the gastrointestinal tract comprise a small but highly bioactive and immunogenic component of the gut microbiota, which can influence liver biology in both direct and indirect ways [38]. On the one hand, fungi cell wall molecules, derived metabolites and secreted peptides can directly translocate across intestinal barriers to the liver and trigger aberrant signaling cascades. On the other hand, certain fungi that inhabit the intestinal mucosa can also transmit signals to the liver through the migration of antigen-reactive cells, despite being distant from the site of their induction. Impaired intestinal barriers are common in patients with liver disease [1], facilitating the dynamics of fungal translocation and changes in the enteric mycobiome to affect the progression of liver disease (Fig 2).

Fungal β-glucans are the most abundant fungal cell wall polysaccharides known for their metabolic and immunomodulatory properties [39]. Chronic alcohol feeding increased the number of fungi in the intestine and the translocation of fungal β-glucans to the systemic circulation [23]. C-type lectin domain family 7 member a (Clec7a; also known as dectin1) is a pattern recognition receptor that recognizes β-glucans. Upon binding to Clec7a on Kupffer cells, β-glucans activated caspase-1 via NLR Family Pyrin Domain Containing 3 (NLRP3) that leads to increased inflammatory Interleukin (IL)-1b expression and secretion, which subsequently contributed to hepatocyte damage and ethanol-induced liver disease. However, a recent study suggested a protective role of *Candida*-derived β-glucan in lipopolysaccharide and D-galactosamine-induced hepatitis [40]. Pretreatment with the β-glucan 9 h before the LPS/D-GalN challenge markedly reduced LPS/D-GalN-induced mortality in mice via activating/cleaving enzyme Adam-17 expression and serum Tumor Necrosis Factor Soluble Receptor 1 (TnfsR-1). This discrepancy may be due to different liver diseases and due to the difference in the exposure time of fungal β-glucans. Long-term exposure to β-glucan continuously activates the cellular inflammasome pathway while short-term exposure induces anti-apoptotic signaling [40].

Fungus-derived prostaglandins are oxylipins known as important lipid mediators of inflammation [41]. Gut fungi-induced prostaglandin E2 (PGE2) production in the liver increased hepatic fat accumulation and inflammatory injury in mice with ethanol-induced liver disease [42]. Hepatocyte PGE2 receptors EP2 and EP4 were also up-regulated after chronic ethanol feeding. While inhibition of gut fungi or PGE2 synthase significantly inhibited hepatic PGE2, EP2, EP4, and chemokine (C-X-C motif) ligand 1 (CXCL1), and reduced the development of alcohol-associated hepatic steatosis. Some evidence showed that PGE2 can also be produced by resident liver macrophages, called Kupffer cells, after stimulation with LPS [43]. Therefore, it is sometimes difficult to distinguish fungi-produced PGE2 from fungi-activated hepatic PGE2 production. PGE2 also contributed to the development of steatosis by enhancing lipid accumulation in the liver and suppressing VLDL synthesis and β-oxidation [44,45]. Therefore, PGE2 could be involved in the development of fatty liver diseases via multiple ways.

Candidalysin is a secreted peptide by *C*. *albicans* and it is known for its cytolytic activity [46]. Candidalysin causes direct hepatocyte damage and promotes alcohol-associated liver disease. Extent of cell elongation 1 (*ECE1*), the gene encoding candidalysin in *C*. *albicans*, was assessed via qPCR of human stool samples. The percentages of patients carrying *ECE1* was increased and positively correlated with the severity of disease in patients with alcohol-associated hepatitis [25]. Patients with fecal samples tested “*ECE1*-positive” had increased gut permeability, higher MELD (model for end-stage liver disease) score and 90-day mortality compared with “*ECE1*-negative” patients, suggesting that candidalysin produced in the intestinal lumen could reach the liver via increased intestinal permeability and exert its effects on the liver. In addition to its effects on hepatocyte damage, candidalysin was also reported to activate mitogen-activated protein kinase (MAPK) signaling [47] and the NLRP3 inflammasome [48], which contributed to the pathophysiology of NAFLD [49,50]. Since the increase of *C*. *albicans* was associated with the progression of NAFLD, future studies should address the role of candidalysin for NAFLD.

In addition to the direct disease-promoting effects of translocating bioactive fungi-derived components, fungi-primed immune cells can also migrate to peripheral organs and potentially affect liver disease progression. Our group discovered that patients with alcohol use disorder and liver disease had specifically increased peripheral and hepatic *C*. *albicans*-specific Th17 cells [51]. We confirmed the migration of *C*. *albicans*-specific Th17 cells from the intestine to the liver in mice where they contributed to ethanol-induced liver disease via the secretion of IL-17. Hepatic IL-17 bound to IL-17 receptors on Kupffer cells to induce inflammatory IL-1b production. The increased fungi-induced immune response is a double-edged sword, which protects against fungal translocation at barrier sites, but migration of these cells to the liver causes damage via chronic stimulation by translocated fungal products.

Aside from the liver, there is evidence that the gut microbiota regulates the migration of lymphocytes from the gut to the lung, brain, and kidney. Intestinal inflammation expanded *C*. *albicans*-specific and cross-reactive Th17 cells can contribute to lung inflammation induced by airborne *A*. *fumigatus* [52]; *segmented filamentous bacteria* (SFB)-induced gut Th17 cells were preferentially recruited to the lung by the Th17 chemoattractant, C-C motif ligand 20 (CCL20), to distantly provoke lung pathology [53]. Intestinal dysbiosis increased regulatory T cells and decreased IL-17^+^γδ T cells through altered dendritic cell activity, which suppressed the trafficking of effector T cells from the gut to the leptomeninges after a stroke [54]. Intestinal Th17 cells contributed to autoimmune kidney disease via the CCL20/CC chemokine receptor type 6 (CCR6) axis and in an S1P-receptor-1-dependent fashion [55].

Exposure to fungi and their products could also shape the host antibody repertoire and affect extraintestinal tissues. Antibodies against fungal β-glucan and other fungal antigens detected in the serum of healthy individuals, were increased in patients with various liver diseases and correlated with disease severity, including in patients with PSC [56], alcohol-associated cirrhosis [23], alcohol-associated hepatitis [22], and alcohol use disorder [24]. Anti-*Saccharomyces cerevisiae* antibodies (ASCA) and anti-*Candida albicans* antibodies are two of the most important markers in liver disease. Doron and colleagues used a multi-kingdom antibody profiling (multiKAP) approach and found that human antibody repertoires against gut mycobiota depend on the innate immunity regulator CARD9. Furthermore, *Candida albicans* was identified as the major inducer of anti-fungal IgG [57]. However, induction and regulation of anti-fungal antibodies, and their relevance for the development of liver disease requires future research studies.

### Intrahepatic mycobiome in liver diseases

Gut fungal products such as 1,3-β-D-glucan reach the systemic circulation, although the translocation path (paracellular diffusion, transcellular transport, or co-transport with chylomicrons) is not well understood. The question arises whether viable fungi translocate to the liver and affect liver disease? Liver is generally regarded as a sterile organ that does not harbor microbes [58]. Several recent studies reported the presence of intrahepatic bacteria in healthy individuals [59], patients with NAFLD [60], mouse models of ethanol-induced liver disease liver disease [61] and autoimmune hepatitis [62]. Gut bacteria can be selectively introduced to the liver, undergo dynamic changes in response to the host physiology, and actively affect, and in some cases predict the progression of liver disease [63]. Matched liver and fecal specimens suggested increased bacterial DNA load, lower bacterial alpha diversity, and higher *Proteobacteria* in the livers of patients with NAFLD compared with healthy controls [59]. Several intrahepatic bacteria such as *Bacteroidetes* species [64] and *Lactobacillus reuteri* [62] were found to contribute to hepatic immunity through their metabolic byproducts, e.g., glycosphingolipid and indole-3-aldehyde that potentiate liver inflammatory cell recruitment and maturation via NKT cell activation or Interferon (IFN)-gamma producing CD8 T cells.

Recent studies shed light on the characterization of fungal translocation to multiple body sites, including tumor and normal adjacent tissue of breast, lung, melanoma, ovary, colon, brain, bone, and pancreas using an array of diverse techniques [65]. *Candida*, *Saccharomyces*, *Malassezia*, *Blastomyces*, and *Cladosporium* were present within cancer cells and macrophages in various tumor tissues. Intra-tumoral fungal communities exhibited cancer type-specific profiles and drive distinct immune responses together with bacterial communities that stratify patient survival. Moreover, several studies establish causal relationships between intra-tumor fungi and tumor growth. Anti-fungal treatment significantly decreased intra-tumor fungi and pancreatic ductal adenocarcinoma tumor burden, increased survival via IL-33 secretion and suppressed recruitment of type 2 innate lymphoid cells (ILC2) and Th2 cells in the tumor microenvironment [66]. On the other hand, *Malassezia globosa* or *Alternaria alternata* administration augmented the infiltration of ILC2 and promoted tumor growth [66]. In another study, *Malassezia globosa* was shown to migrate from the gut lumen to the pancreas, activating the complement cascade through the activation of mannose-binding lectin that was required for oncogenic progression [67]. These studies establish causal relationships between intra-tissue fungi and disease progression, indicating that fungi are sparse but immunologically potent, thus raising the possibility to explore fungal translocation to the liver and intrahepatic mycobiome.

However, it is challenging to establish the existence of a functional fungal niche in liver due to the low biomass. Researchers have used ITS2 amplicon sequencing, whole genome sequencing and quantitative polymerase chain reaction of the fungal 5.8S ribosomal gene to identify the existence of fungal nucleic acids in different tissue types. Fungi can also be detected by multiple staining methods within tissues including a fungal cell wall-specific anti-β-glucan antibody, anti-*Aspergillus* antibody, fluorescence in situ hybridization (FISH) against fungal 28S rRNA sequences and fungal cell wall-specific Gomori methenamine silver (GMS) stain [65]. Despite the use of complementary strategies and sterile tissue protocols to avoid the false-positive signal, there is an ongoing debate on the technical challenges analyzing fungal sequencing data and their association with disease progression from low-biomass samples [68]. In addition, future studies are required to determine whether intra-organ fungi are viable, can be cultured, and remain metabolically active to affect the progression of liver disease.

## Conclusions and perspectives

The integration of high-throughput sequence-based technologies with detailed mechanistic investigations has significantly broadened our understanding of how the gut mycobiome influences the physiology and pathology of liver diseases. Alterations in the composition of fungal communities and translocation of fungal products from the gut to the liver play a role in the initiation and progression of liver diseases. Gut fungi are recognized for their intricate life cycles that can switch from unicellular to multicellular, yet the impact of strain variation and fungal evolution in the gut on liver disease remains unclear. Despite being in its early stages, manipulation of the gut mycobiome has demonstrated promising therapeutic effects on liver diseases. The use of antifungal drugs or probiotics derived from fungi has proven effective in the treatment of various liver diseases in preclinical models, although the translation of these results to human subjects is still pending. To further advance gut mycobiome-based therapies for liver disease, more precise approaches targeting specific fungi or functional fungal genes are necessary.

## Supporting information

S1 Table Alterations of gut mycobiome in liver disease. (DOCX)
