# Supplementary material for: Gut mycobiome alterations and implications for liver diseases
Source: PLoS Pathog. 2024 Aug 8;20(8):e1012377. doi: 10.1371/journal.ppat.1012377 (PMC11309506; doi:10.1371/journal.ppat.1012377)
Supplement: S1 Table — (DOCX) [file ppat.1012377.s001.docx]

**S1 Table. Alterations of gut mycobiome in liver disease.**

| **Cohorts** | **Country** | | **Diversity** | **Method** | | **Compositional change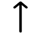** | **Compositional change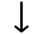** | **Ref.** |
| --- | --- | --- | --- | --- | --- | --- | --- | --- |
| **Metabolic dysfunction-associated fatty liver disease (MAFLD)** | | | | | | | | |
| NAFLD (n = 79)  Controls (n = 34) | China | **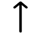** | | | ITS2 | *Talaromyces*  *Paraphaeosphaeria*  *Lycoperdon*  *Curvularia*  *Phialemoniopsis*  *Paraboeremia*  *Sarcinomyces*  *Cladophialophora*  *Sordaria* | *Leptosphaeria*  *Pseudopithomyces*  *Fusicolla* | [1] |
| MAFLD (n = 21)  Controls (n = 20) | China | 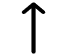 | | | Metagenomics | *Mucor ambiguus* |  | [2] |
| NAFLD (n = 79)  AUD (n = 73)  Controls (n = 16) | Germany | N. S. | | | ITS2 | Log-ratio: *Mucor sp./Saccharomyces cerevisiae* |  | [3] |
| **Alcohol-associated liver disease** | | | | | | | | |
| Alcoholic hepatitis (n =59)  AUD (n = 15)  Controls (n = 11) | USA | 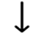 | | | ITS1 | *Candida* | *Penicillium* | [4] |
| AUD (n = 10)  Alcohol- associated hepatitis (n = 6)  Alcohol- associated cirrhosis (n = 4)  Controsl (n = 8) | China | 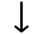 | | | ITS1 | *Candida* | *Epicoccum*  *Galactomyces*  *Debaryomyces* | [5] |
| AUD (n = 66)  Controls (n = 18) | Belgium | Not reported | | | ITS2 | *Candida albicans*  *Candida zeylanoides*  *Issatchenkia orientalis*  *Scopulariopsis cordiae* | *Kazachstania humilis* | [6] |
| Alcohol -associated hepatitis (n = 91)  AUD (n = 42)  Controls (n = 91) | Multi-center | Not reported | | | Culture and single colony qPCR | *Candida albicans* |  | [7] |
| AUD (n = 66)  Controls (n = 18) | Belgium | Not reported | | | ITS2 | *Malassezia restricta* |  | [8] |
| **Primary sclerosing cholangitis** | | | | | | | | |
| PSC (n = 65)  UC (n = 38)  Controls (n = 66) | Germany | N.S. | | | ITS2 | *Candida*  *Humicola griseum* |  | [9] |
| PSC with UC (n = 26)  UC (n = 27)  Controls (n = 26) | Italian | N.S. | | | ITS2 | *Saccharomyces*  *Sporobolomyces*  *Tilletiopsis*  *Debaryomyces* |  | [10] |
| PSC with IBD (n = 27)  PSC without IBD (n = 22)  IBD (n = 33)  Controls (n = 30) | French | 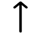 | | | ITS2 | *Exophiala* | *Saccharomyces cerevisiae* | [11] |
| **Cirrhosis and Hepatocellular carcinoma** | | | | | | | | |
| Cirrhosis inpatient (n = 66)  Cirrhosis outpatient (n = 77)  Controls (n = 26) | USA | 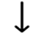 | | | ITS1 | *Candida* |  | [12] |
| HCC (n = 34)  Cirrhosis (n = 20)  Control (n = 18) | China | 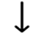 | | | ITS2 | *Malassezia*  *Malassezia sp.*  *Candida*  *C. albicans* |  | [13] |
| Cirrhosis patients (n = 135)  Control (n = 26) | Germany | Not reported | | | Culture  PCR | *C. albicans* |  | [14] |

Abbreviations: MAFLD, Metabolic dysfunction-associated fatty liver disease; NAFLD, non-alcoholic fatty liver disease; AUD, alcohol use disorder; PSC, primary sclerosing cholangitis; UC, ulcerative colitis; IBD, inflammatory bowel disease; HCC, hepatocellular carcinoma; N.S., no significant difference; ITS, internal transcribed space

References

1. You N, Xu J, Wang L, Zhuo L, Zhou J, Song Y, et al. Fecal Fungi Dysbiosis in Nonalcoholic Fatty Liver Disease. Obesity. 2021;29: 350–358. doi:10.1002/oby.23073

2. Niu C, Tu Y, Jin Q, Chen Z, Yuan K, Wang M, et al. Mapping the human oral and gut fungal microbiota in patients with metabolic dysfunction-associated fatty liver disease. Front Cell Infect Microbiol. 2023;13: 1157368. doi:10.3389/fcimb.2023.1157368

3. Demir M, Lang S, Hartmann P, Duan Y, Martin A, Miyamoto Y, et al. The fecal mycobiome in non-alcoholic fatty liver disease. Journal of Hepatology. 2022;76: 788–799. doi:10.1016/j.jhep.2021.11.029

4. Lang S, Duan Y, Liu J, Torralba MG, Kuelbs C, Ventura‐Cots M, et al. Intestinal Fungal Dysbiosis and Systemic Immune Response to Fungi in Patients With Alcoholic Hepatitis. Hepatology. 2020;71: 522–538. doi:10.1002/hep.30832

5. Yang A-M, Inamine T, Hochrath K, Chen P, Wang L, Llorente C, et al. Intestinal fungi contribute to development of alcoholic liver disease. Journal of Clinical Investigation. 2017;127: 2829–2841. doi:10.1172/JCI90562

6. Hartmann P, Lang S, Zeng S, Duan Y, Zhang X, Wang Y, et al. Dynamic Changes of the Fungal Microbiome in Alcohol Use Disorder. Front Physiol. 2021;12: 699253. doi:10.3389/fphys.2021.699253

7. Chu H, Duan Y, Lang S, Jiang L, Wang Y, Llorente C, et al. The Candida albicans exotoxin candidalysin promotes alcohol-associated liver disease. Journal of Hepatology. 2020;72: 391–400. doi:10.1016/j.jhep.2019.09.029

8. Zeng S, Hartmann P, Park M, Duan Y, Lang S, Llorente C, et al. Malassezia restricta promotes alcohol-induced liver injury. Hepatol Commun. 2023;7: e0029. doi:10.1097/HC9.0000000000000029

9. Rühlemann MC, Solovjeva MEL, Zenouzi R, Liwinski T, Kummen M, Lieb W, et al. Gut mycobiome of primary sclerosing cholangitis patients is characterised by an increase of Trichocladium griseum and Candida species. Gut. 2020;69: 1890–1892. doi:10.1136/gutjnl-2019-320008

10. Del Chierico F, Cardile S, Baldelli V, Alterio T, Reddel S, Bramuzzo M, et al. Characterization of the Gut Microbiota and Mycobiota in Italian Pediatric Patients With Primary Sclerosing Cholangitis and Ulcerative Colitis. Inflamm Bowel Dis. 2023; izad203. doi:10.1093/ibd/izad203

11. Lemoinne S, Kemgang A, Ben Belkacem K, Straube M, Jegou S, Corpechot C, et al. Fungi participate in the dysbiosis of gut microbiota in patients with primary sclerosing cholangitis. Gut. 2020;69: 92–102. doi:10.1136/gutjnl-2018-317791

12. Bajaj JS, Liu EJ, Kheradman R, Fagan A, Heuman DM, White M, et al. Fungal dysbiosis in cirrhosis. Gut. 2018;67: 1146–1154. doi:10.1136/gutjnl-2016-313170

13. Zhang L, Chen C, Chai D, Li C, Qiu Z, Kuang T, et al. Characterization of the intestinal fungal microbiome in patients with hepatocellular carcinoma. J Transl Med. 2023;21: 126. doi:10.1186/s12967-023-03940-y

14. Krohn S, Zeller K, Böhm S, Chatzinotas A, Harms H, Hartmann J, et al. Molecular quantification and differentiation of Candida species in biological specimens of patients with liver cirrhosis. PLoS One. 2018;13: e0197319. doi:10.1371/journal.pone.0197319
